# Editorial: Novel Applications of Stereotactic Body Radiotherapy (SBRT)

**DOI:** 10.3389/fonc.2021.760078

**Published:** 2021-09-03

**Authors:** Rupesh Kotecha, Debnarayan Dutta, Rakesh Jalali, Alexander Muacevic

**Affiliations:** ^1^Department of Radiation Oncology, Miami Cancer Institute, Baptist Health South Florida, Miami, FL, United States; ^2^Herbert Wertheim College of Medicine, Florida International University, Miami, FL, United States; ^3^Amrita Institute of Medical Sciences and Research Centre, Kochi, India; ^4^Apollo Proton Cancer Centre, Chennai, India; ^5^Ludwig Maximilian University of Munich, Munich, Germany

**Keywords:** stereotactic body radiotherapy, stereotactic ablative body radiation, oligometastatic, oligoprogressive, hepatocellular carcinoma, arteriovenous malformation

The use of stereotactic body radiotherapy (SBRT) or stereotactic ablative radiotherapy (SABR) has undergone a surge in enthusiasm around the world fueled by an improved understanding of the radiobiology of high-fractional dose radiotherapy, technology development and increased availability of high-precision delivery platforms, and the dissemination of published literature through retrospective and prospective studies. Our current knowledge of SBRT/SABR has not only expanded the use of this treatment approach in the historical and classic scenarios of brain and spine SBRT/SABR and lung SBRT/SABR, but also led to new frontiers of clinical applications. In this context, several recent studies were published within a special Research Topic specifically focused on novel and unique applications of SBRT/SABR.

Greve et al. published a large retrospective analysis of patients treated with stereotactic radiosurgery (SRS) for arteriovenous malformations with CT and MRI-based planning approaches and without stereotactically-defined digital subtraction angiography over a 14-year period. In total, this series evaluated the outcomes of 215 patients (53% treated with SRS as a first-line treatment and 55% classified as Spetzler-Martin grade I-III) treated to a median dose of 18 Gy in 1 fraction to a median target volume of 2.4 cm^3^. Approximately 47% demonstrated complete obliteration of the arteriovenous malformation, consistent with that observed in other series using different treatment platforms, and supporting the use of this approach.

Although spine SRS/SBRT has been established as an effective treatment in the upfront management of patients with spinal metastasis, Ehret et al. expand the evidence for treatment of recurrent spine metastasis treated with SRS. In their study of 53 patients (initial treatment 36 Gy in 15 fractions treated to a median dose of 18 Gy in 1 fraction), the local control rate was 77% (Ehret et al.). Furthermore expanding indications from metastatic sites to intramedullary lesions, Ehret et al. report on 12 patients with WHO II/III spinal ependymomas treated to a median dose of 15 Gy in 1 fraction with a local control rate of 84%. Together, these series support the role of spine SRS in patients with extramedullary and intramedullary tumors.

Liu et al. reported on patients with hepatocellular carcinoma, and although radiotherapy was not a particular focus of their study, demonstrate potential avenues for evidence development by guiding patient selection for adjuvant treatment. They report on a series of 244 patients with hepatocellular carcinoma treated with narrow-margin (<1 cm margin) or wide-margin (≥1 cm margin) resections (Liu et al.). In total, post-operative recurrence was observed in 53% of patients and not only was the risk of recurrence higher in those treated with narrow-margin resections, the pattern of recurrence was also different, with modest rates of marginal recurrence (21 vs. 5%). These recurrence patterns were also hypothesized to affect survival as those patients treated with narrow margins also had reduced overall survival. Interestingly, post-operative SBRT for patients with positive margins yielded no marginal recurrences. In addition, Jiang et al. evaluated the safety and efficacy of transarterial chemoembolization (TACE) and SBRT for Barcelona Clinic Liver Cancer (BCLC) stage B hepatocellular carcinoma. In their series, 57 patients were treated to a dose of 20-50 Gy in 3-5 fractions; at a median follow-up of 42 months, the objective response rate was 86% and the disease control rate was 97%. These results will help inform the clinical practice of SBRT with consideration of post-operative SBRT in patients at high-risk for local recurrence after surgery as well as definitive treatment for those patients too high-risk for invasive interventions.

SBRT for prostate cancer is now endorsed by national and international treatment guidelines and Aghdam et al. describe the demographic characteristics of patients treated with SBRT over a 10-year period. Interestingly, in their analysis of 1,035 patients treated with prostate SBRT, travel distance did not adversely affect use in African Americans, elderly patients, or those from rural locations, supporting the broad adoption and utilization of this treatment approach. Ultimately, the convenience of SBRT over conventionally-fractionated regimens allows for improved patient access to care.

The use of SBRT/SABR for patients with oligometastatic or oligoprogressive disease is an area of intense study and recent reports have supported the cost-effectiveness of this treatment strategy (Mehrens et al.). Even in patients with pancreatic cancer with liver-only oligometastatic disease, SBRT in addition to chemotherapy appears safe and effective (Ji et al.). Yet, treatment of lymph nodes with stereotactic radiotherapy remains an understudied area. Burkon et al. reported on a retrospective analysis of 90 patients treated with SBRT to lymph nodes in the mediastinum, retroperitoneum, or pelvis. The local control rate was modest at 69% at 3 years and the median freedom from widespread dissemination was 14.6 months. Additional studies will evaluate the patterns of failure, optimal dose and fractionation schedule, and patient selection criteria for this treatment approach.

As we look towards the future of SBRT/SABR, the introduction of innovative radiotherapy delivery approaches represents a relatively nascent area of study. Feasibility studies have begun to demonstrate the potential of particle therapy delivery techniques, such as Spot-Scanning Proton Arcs (Liu et al.), and future trials will incorporate these novel technologies with increasing clinical indications.

## Author Contributions

All authors have reviewed this manuscript. All authors contributed to the article and approved the submitted version.

## Conflict of Interest

RK: Honoraria from Accuray Inc., Elekta AB, Viewray Inc., Novocure Inc., Elsevier Inc, Brainlab. Institutional research funding from Medtronic Inc., Blue Earth Diagnostics Ltd., Novocure Inc., GT Medical Technologies, Astrazeneca, Exelixis, Viewray Inc, Brainlab.

RJ: Honoraria from Cipla Limited. AM: Honoraria from Accuray Inc.

The remaining author declares that the research was conducted in the absence of any commercial or financial relationships that could be construed as a potential conflict of interest.

## Publisher’s Note

All claims expressed in this article are solely those of the authors and do not necessarily represent those of their affiliated organizations, or those of the publisher, the editors and the reviewers. Any product that may be evaluated in this article, or claim that may be made by its manufacturer, is not guaranteed or endorsed by the publisher.

